# Identification of a new family of enzymes with potential *O*-acetylpeptidoglycan esterase activity in both Gram-positive and Gram-negative bacteria

**DOI:** 10.1186/1471-2180-5-49

**Published:** 2005-08-19

**Authors:** Joel T Weadge, John M Pfeffer, Anthony J Clarke

**Affiliations:** 1 Department of Molecular and Cellular Biology, University of Guelph, Guelph, Ontario N1G 2W1 Canada

## Abstract

**Background:**

The metabolism of the rigid bacterial cell wall heteropolymer peptidoglycan is a dynamic process requiring continuous biosynthesis and maintenance involving the coordination of both lytic and synthetic enzymes. The *O*-acetylation of peptidoglycan has been proposed to provide one level of control on these activities as this modification inhibits the action of the major endogenous lytic enzymes, the lytic transglycosylases. The *O*-acetylation of peptidoglycan also inhibits the activity of the lysozymes which serve as the first line of defense of host cells against the invasion of bacterial pathogens. Despite this central importance, there is a dearth of information regarding peptidoglycan *O*-acetylation and nothing has previously been reported on its de-acetylation.

**Results:**

Homology searches of the genome databases have permitted this first report on the identification of a potential family of *O-*Acetylpeptidoglycan esterases (Ape). These proteins encoded in the genomes of a variety of both Gram-negative and Gram-positive bacteria, including a number of important human pathogens such as species of *Neisseria*, *Helicobacter*, *Campylobacter*, and *Bacillus anthracis*, have been organized into three families based on amino acid sequence similarities with family 1 being further divided into three sub-families. The genes encoding these proteins are shown to be clustered with Peptidoglycan *O*-acetyltransferases (Pat) and in some cases, together with other genes involved in cell wall metabolism. Representative bacteria that encode the Ape proteins were experimentally shown to produce *O*-acetylated peptidoglycan.

**Conclusion:**

The hypothetical proteins encoded by the *pat *and *ape *genes have been organized into families based on sequence similarities. The Pat proteins have sequence similarity to *Pseudomonas aeruginosa *AlgI, an integral membrane protein known to participate in the *O*-acetylation of the exopolysaccaride, alginate. As none of the bacteria that harbor the *pat *genes produce alginate, we propose that the Pat proteins serve to *O*-acetylate peptidoglycan which is known to be a maturation event occurring in the periplasm. The Ape sequences have amino acid sequence similarity to the CAZy CE 3 carbohydrate esterases, a family previously known to be composed of only *O*-acetylxylan esterases. They are predicted to contain the α/β hydrolase fold associated with the GDSL and TesA hydrolases and they possess the signature motifs associated with the catalytic residues of the CE3 esterases. Specific signature sequence motifs were identified for the Ape proteins which led to their organization into distinct families. We propose that by expressing both Pat and Ape enzymes, bacteria would be able to obtain a high level of localized control over the degradation of peptidoglycan through the attachment and removal of *O*-linked acetate. This would facilitate the efficient insertion of pores and flagella, localize spore formation, and control the level of general peptidoglycan turnover.

## Background

**Figure 1:**
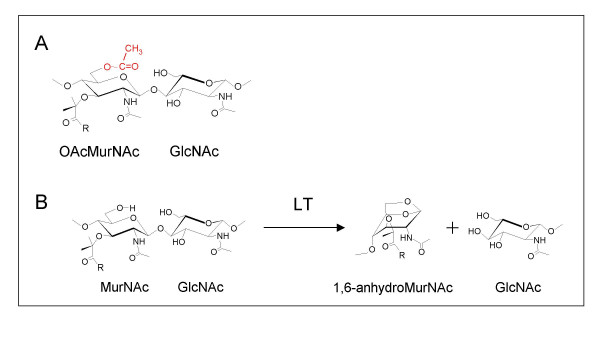
**(A) Structure of *O*-acetylpeptidoglycan and (B) reaction pathway of lytic transglycosylases (LT)**. A. The *O*-acetylation of peptidoglycan occurs at the C-6 hydroxyl group of MurNAc residues (shown in red) to generate the corresponding 2-*N*-6-*O*-diacetylmuramyl residue (OAcMurNAc). B. The lytic transglycosylases (LT) require a free C-6 hydroxyl group to catalyze the formation of 1,6-anhdyroMurNAc products. The R denotes the stem peptide associated with the C-3 lactyl moiety of MurNAc residues.

Peptidoglycan is an essential component of most eubacterial cell walls (the exceptions are the mycobacteria). It provides osmotic stability by serving as an exo-skeleton through which cellular shape is imparted. The tensile strength of this multilayered polymer is provided by two types of covalent linkages. The first consists of β-1,4 glycosidic bonds between N-acetylmuramic acid (MurNAc) and N-acetylglucosamine (GlcNaC), while the second occurs through cross-linking of the tetrapeptides associated with MurNAC residues on adjacent glycan strands (Figure 1).

Peptidoglycan biosynthesis occurs in three stages (reviewed in [1]). The first involves the synthesis of soluble precursors in the cytoplasm leading to the production of UDP-linked MurNAc-pentapeptide. This muropeptide is then transferred to the membrane carrier bactoprenol via a pyrophosphate group and a GlcNAc residue is added to produce the membrane-associated, lipid II precursor. By an unknown mechanism, this lipid-linked precursor is translocated to the outer face of the cytoplasmic membrane. Now exposed to the periplasm in this third stage of biosynthesis, the available muropeptide is polymerized into the existing peptidoglycan sacculus, a reaction catalyzed by the transglycosylase domain of the class A high-molecular weight penicillin-binding proteins (PBPs). Once polymerized, the transpeptidase domain of both the class A and class B high-molecular weight PBPs catalyze the formation of cross-links between the peptides of neighboring glycan strands.

The peptidoglycan sacculus which surrounds the entire bacterium is not a static structure as it must permit the growth and division of the bacterial cell [1]. Furthermore, insertion of cell wall structures that transverse the peptidoglycan layer, such as secretion/transport complexes, flagella, and pili, require the sacculus to be constantly remodeled and reinforced [2]. These processes are performed by the coordinated action of peptidoglycan synthesizing PBPs and the peptidoglycan lytic enzymes. The peptidoglycan cleaving enzymes are numerous and there exists a specific lytic activity for each linkage in peptidoglycan. The amidases cleave the stem peptide from the MurNAc residues, glucosaminidases hydrolyze the β-1,4 linkage between GlcNAc and MurNAc residues, and both the lytic transglycosylases and muramidases cleave the other β-1,4 glycosidic bond that exists between MurNAc and GlcNAc (reviewed in [3]). Collectively, these enzymes are called autolysins because their uncontrolled activity will lead to the lysis of bacterial cells. Given the potential lethal activity of the autolysins, the cell has to control their activity. This is accomplished, at least in part, by associating these enzymes in a holoenzyme complex that includes both the lytic enzymes, which provide sites for the insertion of new peptidoglycan precursors, and the synthesizing PBPs. The major glycolytic enzyme associated with these holoenzyme complexes appears to be the lytic transglycosylases [1]. These enzymes are not hydrolases but cleave the β-1,4 linkage between GlcNAc and MurNAc with the concomitant formation of 1,6-anhydromuramoyl residues [4] (Fig. 1).

Given the inherent importance of peptidoglycan, it is not surprising that lysozyme, one of the most important components of the first line innate immune response of eukaryotic hosts to invading bacteria, targets this polymer. Hydrolysis of the β-1,4 glycosidic linkage between GlcNAc and MurNAc destabilizes the integrity of the sacculus and ultimately leads to cell lysis. A number of pathogenic bacteria are able to avoid the potentially lethal effects of lysozyme through the addition of an acetate group to the C-6 position of MurNAc (reviewed in [5,6]) (Figure 1). These pathogens include both Gram-positive and Gram-negative bacteria, such as *Staphylococcus aureus *and *Neisseria gonorrhoeae*, respectively. Several studies have shown that a direct correlation exists between the extent of *O*-acetylation and susceptibility to lysozyme-catalyzed hydrolysis of peptidoglycan [7-11]. However, the *O*-acetylation of peptidoglycan has also been shown to preclude the action of the endogenous lytic transglycosylases [12]. Intuitively, this would be expected given that the mechanism of action of these latter enzymes requires a free C-6 hydroxyl group on MurNAc to produce their reaction product, the corresponding 1,6-anhydro derivative. This has prompted the proposal of two roles for peptidoglycan *O*-acetylation; one to protect the bacterium from a host immune response, while the second would be to regulate the action of endogenous autolysins [13].

There is a dearth of information concerning the pathway for the *O*-acetylation of peptidoglycan, and nothing is known about its subsequent removal. All available evidence indicates that *O*-acetylation is a maturation event, occurring in the periplasm after the cross-linking of newly incorporated muropeptides (reviewed in [5,6]). Two models have been proposed to account for the transfer of acetate from cytoplasmic pools of acetyl-CoA to muramoyl residues in the peptidoglycan sacculus [6]. The first is a two protein system involving an integral membrane protein responsible for the translocation of acetate from the cytoplasm to the periplasm where it is accepted by a second more soluble protein which transfers it specifically to the C-6 hydroxyl of muramoyl residues in peptidoglycan. This system is modeled on the proposed pathway for the *O*-acetylation of the exopolysaccharide, alginate, produced by the opportunistic human pathogen *Pseudomonas aeruginosa *[14,15]. The other model invokes a single-enzyme system, exemplified by the action of Nod X for the *O*-acetylation of the carbohydrate-based nodulation factor by *Rhizobium leguminosarum *[16], which functions to concomitantly translocate and transfer the acetate from the cytoplasm to peptidoglycan. Recently, an enzyme has been identified within *S. aureus *that appears to function according to the second pathway [17], but more experimental evidence will be required to bear this out.

Herein, we describe the identification of a putative cluster of ORFs that encode both hypothetical *O*-acetyltransferases and *O*-acetyl esterases that we propose act on peptidoglycan to provide a dynamic process for attachment and removal of *O*-acetate, respectively. This proposal is supported by experimental data which demonstrate that representative bacteria harboring these ORFs do *O*-acetylate their respective peptidoglycan sacculi. This study thus represents the first to identify the presence of *O*-acetylpeptidoglycan in a variety of other human pathogens and the proteins involved in both the attachment and removal of this *O*-linked acetate.

## Results and discussion

### Initial identification of a peptidoglycan *O*-acetylation cluster in *N. gonorrhoeae*

**Figure 2:**
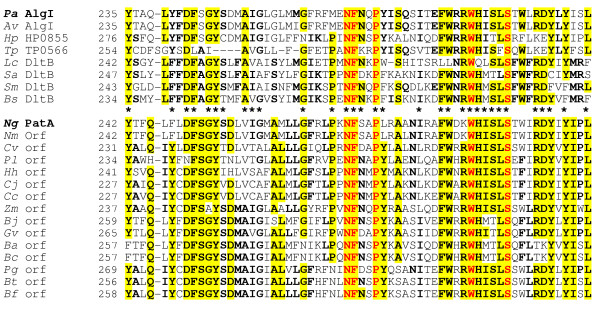
**Sequence alignment of *N. gonorrhoeae *PatA and related hypothetical proteins found in the genome databases with family 1 *O*-acetyltransferases**. The family of proteins presented in the upper block of sequences includes AlgI from both *P. aeruginosa *PAO1 and *Azotobacter vinelandii*, a transporter of acetate involved in the *O*-acetylation of the exopolysaccharide alginate [6]. The residues in bold face and yellow highlight denote greater than 50% and 80% identity, respectively, within the separate blocks of sequences. The asterisks identify conservation between the two blocks of sequences while the residues in red are invariant amongst all sequences. Abbreviations (accession numbers): *Pa*, *P. aeruginosa *(U50202); *Av*, *Azotobacter vinelandii*, (AF027499); *Hp*, *Helicobacter pylori *(AE000596); *Tp*, *Treponema pallidum *(AE001232); *Lc*, *Lactobacillus casei*, (P35855); *Sa*, *Staphylococcus aureus *(D86240); *Sm*, *Streptococcus mutans *(AF049357); *Bs*, *Bacillus subtilis *(P39580), *Ng*, *N. gonorrhoeae *(AAW89272); *Nm*, *N. meningitidis *(NP284200); *Cv*, *Chromobacterium violaceum *(NP903736); *Pl*, *Photorhabdus luminescens *(NP927855); *Hh*, *Helicobacter hepaticus *(NP860615); *Cj*, *Campylobacter jejuni*, (NP281794); *Cc*, *Campylobacter coli *(ZP00366780); *Zm*, *Zymomonas mobilis *(YP162181); *Bj*, *Bradyrhizobium japonicum *(NP767015); *Gv*, *Gloeobacter violaceus *(NP923893); *Ba*, *Bacillus anthracis *(NP843401); *Bc*, *B. cereus *(NP977299); *Pg*, *Porphyromonas gingivalis *(NP905383); *Bt*, *Bacteroides thetaiotaomicron *(NP811593); *Bf*, *Bacteroides. fragilis *(YP101412).

*N. gonorrhoeae *is known to *O*-acetylate its peptidoglycan [5,6], however, the genes responsible for this process remain unidentified. Based on the available data, we have postulated that peptidoglycan *O*-acetylation may follow a similar pathway to that involved with the *O*-acetylation of the exopolysaccharide alginate produced by *Pseudomonas aeruginosa *[6]. Thus, to help identify potential genes associated with *O*-acetylation, the *N. gonorrhoeae *genome was probed with *algI *(U50202) [14,15], a *P. aeruginosa *gene encoding a putative membrane-bound, family 1 *O*-acetyltransferase [6]. A homologue of AlgI with 33% amino acid identity (51% similarity) was discovered in the raw sequence data of the unfinished genome project of *N. gonorrhoeae *FA1090 [18] even though this bacterium does not produce alginate. The ORF encodes 478 amino acids and sequence alignments revealed that the hypothetical protein possesses the signature motifs associated with the family 1 *O*-acetyltransferases [6], including the common F-W-R/N-R-W-H motif in the central region of the proteins (Fig. 2). Given that *N. gonorrhoeae *does not produce alginate, we propose that the hypothetical membrane-protein functions to *O*-acetylate peptidoglycan which is known to occur as a periplasmic, maturation event [5]. Thus, we have named the gene *patA *for peptidoglycan *O*-acetyltransferase A.

**Figure 3:**
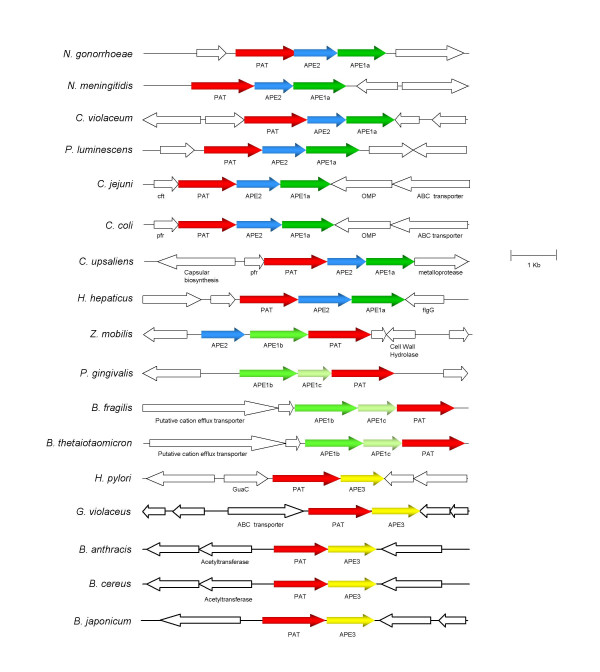
**Gene organizations of *O*-acetylpeptidoglycan (OAP) cluster composed of *pat *and *ape *genes**. Abbreviations for other gene products: cft, ferritin; pfr, ferritin; flgG, flagellar distal rod protein; GuaC, GMP reductase.

**Figure 4:**
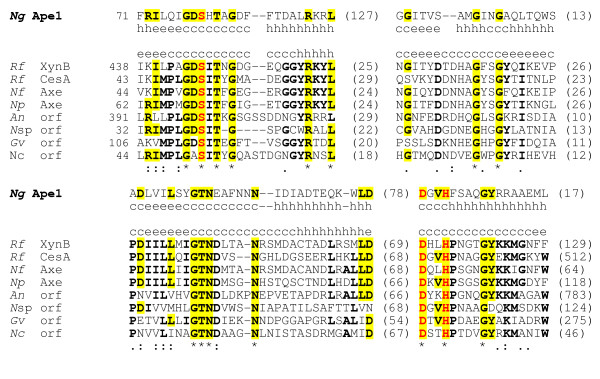
**Sequence alignment and secondary structure predictions of *N. gonorrhoeae *Ape1 and CAZy CE3 carbohydrate esterases (*O*-acetylxylan esterases)**. The residues in bold face denote at least 50% identity amongst the CE-3 esterases, while those highlighted in yellow denote identity with *N. gonorrhoeae *Ape1. Invariant residues are identified by the asterisks and the putative catalytic residues are in red. The numbers in brackets denote the length of intervening sequences. Abbreviations (accession numbers): *Pa*, *P. aeruginosa *(YP207682); *Rf*, *Ruminococcus flavefaciens *(XynB, Q52753; CesA, Q9RLB8); *Nf*, *Neocallimastix frontalis *(U66253); *Np*, *N. patriciarum *(O13497); *An*, *Aspergillus nidulans*, (EAA66937); *Nsp*, *Nomomuraea species *(Q7WZ50); *Gv*, *Gloeobacter violaceus *(Q7NGX3); *Nc*, *Neurospora crassa *(XM323878).

Analysis of sequences downstream from the *patA *locus led to the discovery of two more ORFs which, when subjected to BLASTP sequence similarity searches, permitted their tentative assignment of function to be associated also with the metabolism of acetate (Fig. 3). Thus, the third ORF in this cluster is predicted to be a hypothetical 44,087 Da protein of 397 amino acids having 49% sequence similarity to the catalytic domain of an acetyl xylan esterase (EC 3.1.1.72) from *Ruminococcus flavefaciens *(Fig. 4). This *R. flavefaciens *enzyme belongs to the family 3 carbohydrate esterases (CE3) of the CAZY classification [19]. Each of the other previously known members of family CE3 are also acetyl xylan esterases which aid in the degradation of xylan by removing the blocking O-linked acetate (reviewed in [20]). Although not well characterized, sequence similarity comparisons have permitted the identification of potential catalytic Asp, His, and Ser residues which could form a catalytic triad characteristic of the GDSL and TesA hydrolases [21]. As seen in Figure 4, these invariant residues are also present in the hypothetical *N. gonorrhoeae *protein together with associated signature motifs. Given the significant sequence similarity, it follows that the *N. gonorrhoeae *protein also has similar secondary structural elements compared to the family CE3 proteins, as predicted by the Garnier and PSI PRED programs (Fig. 4).

As *N. gonorrhoeae *is not a xylanolytic bacterium and its genome does not appear to encode xylanases, we postulate that the hypothetical family CE3 esterase functions on peptidoglycan to specifically catalyze its de-*O*-acetylation. This hypothetical protein was thus named Ape1 for *O*-acetylpeptidoglycan esterase 1. It has a high predicted pI value of 8.782 which is typical of periplasmic proteins associated with the metabolism of bacterial cell walls and thus provides further evidence in support of its proposed function. It should be noted that Ape1 (and all of the Ape proteins described below) do not have any significant sequence homology to either the *Streptococcus pneumoniae *peptidoglycan *N*-acetylglucosamine deacetylase (EC 3.1.1.-) [22] or the recently discovered *N*-acetylmuramic acid deacetylase (EC 3.5.1.-) from *Bacillus subtilis *[23], each of which belong to family CE4.

BLASTP analysis of the second ORF in this potential cluster of genes suggested that its deduced sequence of 335 amino acids is weakly similar to lysophospholipase and related esterases (E value 4e-04). Searches for known domains within its sequence using the CD-Search program indicated that it too resembled the GDSL and TesA hydrolases despite having only 18.6% identity with Ape1. Notwithstanding this low identity, the alignment revealed 53.4% similarity between the two hypothetical proteins and further analysis indicated that the second hypothetical protein does possess some of the Ape1 sequence motifs, including the GD**S **and **D**x(V/T)**H **motifs containing the potential catalytic Ser, Asp, and His residues, respectively. Hence, this ORF was tentatively labeled *ape2*.

The *patA, ape1*, and *ape2 *gene sequences discovered on the *N. gonorrhoeae *chromosome were used in BLASTP searches of both the finished and unfinished genome sequences of the NCBI database. These searches led to the identification of 18 other bacteria that possess this cluster of genes (Fig. 3). This list includes some important human pathogens, both Gram-negative and Gram-positive, such as *Campylobacter jejuni*, *Helicobacter pylori*, and *Bacillus anthracis*, respectively.

### Homology of Pat sequences

The recognition of homologs to the acetyltransferase AlgI from *P. aeruginosa *and their organization into a family has been described previously [6]. In the current study, we extend the list with new family members that include sequences from a variety of human pathogens such as species of *Helicobacter*, *Campylobacter*, *Neisseria*, and *Bacillus*, in particular, *Bacillus anthracis *(Fig. 2). All of the additional sequences from these diverse bacteria have at least 49% amino acid similarity to the *P. aeruginosa *AlgI despite the fact that none produce the exopolysaccharide alginate. In addition, each of these sequences contain the membrane-bound O-acetyltransferase (MBOAT) domain (pfam03062) [24]. However, none of the hypothetical proteins have significant homology to the acyltransferase 3 domain (pfam 01757) in the recently identified peptidoglycan *O*-acetyltransferase, OatA, from *Staphylococcus aureus *[17].

### Architecture of the hypothetical *O*-acetylpeptidoglycan esterases (Ape)

**Figure 5:**
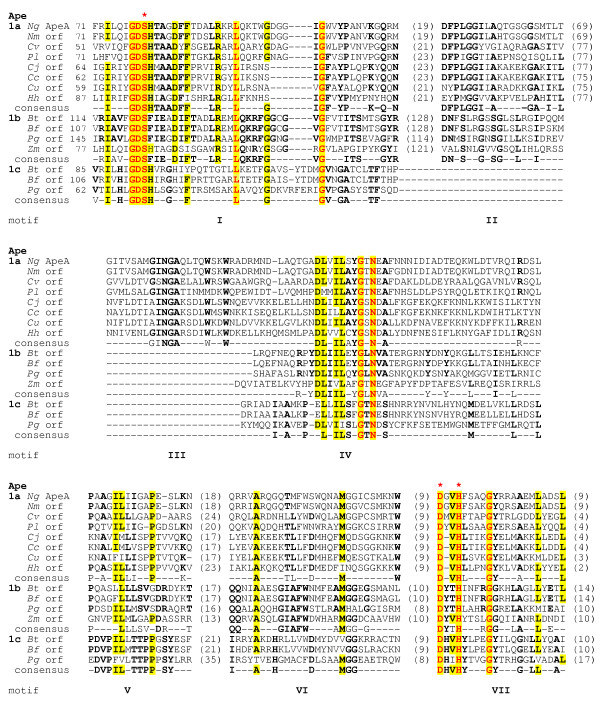
**Sequence alignment of family 1 *O*-acetylpeptidoglycan esterases (Ape) and identification of consensus motifs**. The residues in bold face and yellow highlight denote greater than 50% identity amongst the subfamilies (1A, 1B, 1C) and the entire family, respectively, while those in red are invariant amongst all sequences. The numbers in brackets denote the length of intervening sequences and the potential catalytic residues are identified with the red asterisks. Abbreviations (accession numbers): *Ng*, *N. gonorrhoeae *(AAW89270); *Nm*, *N. meningitidis *(NP284202); *Cv*, *Chromobacterium violaceum *(NP903734); *Pl*, *Photorhabdus luminescens *(NP927853); *Cj*, *Campylobacter jejuni*, (NP281792); *Cc*, *Campylobacter coli *(ZP00366778); *Cu*, *Campylobacter upsaliensis *(ZP00369960); *Hh*, *Helicobacter hepaticus *(NP860613); *Bt*, *Bacteroides thetaiotaomicron *(1b, NP811595; 1c, NP811594); *Bf*, *Bacteroides. fragilis *(1b, YP101414; 1c, NP811594); *Pg*, *Porphyromonas gingivalis *(1b, NP905385; 1c, NP905384); *Zm*, *Zymomonas mobilis *(1b, YP162180).

**Figure 6:**
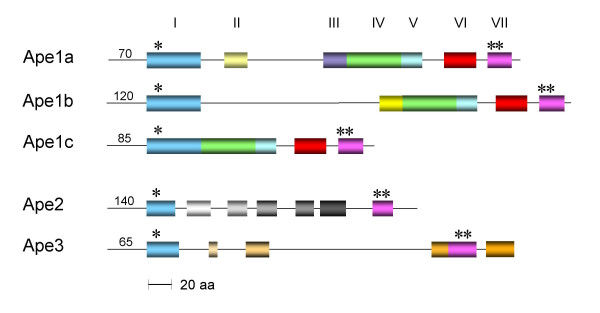
**Distribution of consensus sequence motifs within the three families of Ape proteins**. The motifs of the family 1 Apes are colour coded, and the asterisks denote the locations of the catalytic serine and aspartyl/histidyl residues of motifs I and VII, respectively. The numbers at the beginning of each sequence denote the length of the respective N-terminal sequences.

The *in silico *identification of signature sequence motifs within the hypothetical Ape proteins was performed using a variety of programs. Predictions made using PSI PRED indicated that the sequence of secondary structural elements was very similar amongst each of the deduced proteins and that each contained the α/β hydrolase fold associated with the GDSL and TesA hydrolases (data not shown). This analogy was extended using Clustal W alignments and MEME/MAST motif discovery searches which demonstrated the strict conservation of two signature motifs that are also associated with the GDSL and TesA hydrolases (Fig. 5). The first of these, motif I, is characterized by the sequence GD**S**(H/F) which contains the potential catalytic serine residue, while the **D**x(V/T)**H **of the other (motif VII) includes both the catalytic aspartyl and histidyl residues. In addition to these common sequence motifs, unique signature motifs were discerned within the intervening regions of the hypothetical proteins which permitted the subdivision of the individual sequences into a total of three separate Ape families, with family 1 being further divided into subfamilies (labeled a, b, and c) (Fig. 6). While the intervening sequences motifs distinguish the three Ape families, they nonetheless maintain the overall α/β fold of the proteins.

The Ape1 family of proteins are characterized by three additional common segments of sequence similarity which exist toward the C-terminal end of the proteins and thus closer to motif VII (Figs. 5 and 6). Three respective consensus motifs, labeled IV, V, and VI, were identified within these regions and hence, the Ape1 sequences are characterized by a total of five common sequence motifs.

The subfamily classification of the family 1 proteins is based on the presence or absence of a large sequence of approximately 100 amino acids immediately following motif I. Ape1a and Ape1b subfamilies contain this segment while it is absent in the family 1c sequences. Within this segment, only one conserved motif could be identified (motif II) and differences within it delineate the two subfamilies 1a and 1b. Although the rest of this extra segment is not highly conserved amongst the Ape1a and 1b homologs, a high amount of β-sheet structure is predicted for each. In an attempt to identify its possible function, the entire segment from each of the 12 hypothetical proteins was subjected to BLASTP searches. The only known protein that could be retrieved with any significance (E ≥ 1.0) from the database by this search was penicillin-binding protein 4 (PBP 4) from *Bacillus subtilis *which was obtained by probing with the segment from *B. thetaiotamicron *Ape1b. Interestingly, amino acid sequences similar to this N-terminal region of *B. subtilis *PBP4 are found in the N-terminal regions of a variety of enzymes related to peptidoglycan metabolism, including *S. aureus *PBP4, *Streptocococcus pneumoniae *PBP3, *Streptomyces *K15 D-transpeptidase, *Escherichia coli *PBP5, and TEM-1 β-lactamase. In *S. aureus *PBP4 [25] and *Streptococcus pneumoniae *PBP3 [26] (1TVF and 1XP4, respectively), the homologous sequences comprise a lobe of four surface exposed α-helices as part of the N-terminal, D,D carboxypeptidase-like domain. These helices are positioned on the back side of the active site cleft of the respective enzymes and perhaps participate in protein-protein interactions that contribute to the formation of peptidoglycan biosynthetic complexes.

The family 1a sequences are characterized by the presence of an additional region of sequence homology which is unique to these proteins. Amongst the residues that comprise the consensus motif (motif III) identified within this segment are two of the relatively rare tryptophan residues present in these proteins. This segment may thus contribute to the proteins binding specificity given the prominent role that tryptophanyl residues play in the binding subsites of carbohydrate-active enzymes through stacking interactions.

**Figure 7:**
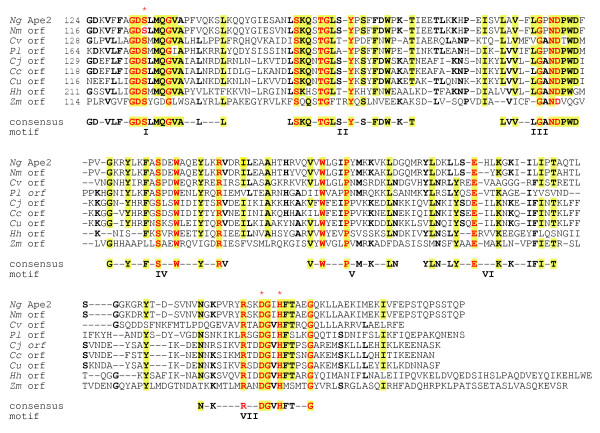
**Sequence alignment of family 2 *O*-acetylpeptidoglycan esterases (Ape2) and identification of consensus motifs**. The residues in bold face and yellow highlight denote greater than 50% and 80% identity, respectively, while those in red are invariant amongst all sequences. The numbers in brackets denote the length of intervening sequences and the potential catalytic residues are identified with the red asterisks. Abbreviations (accession numbers): *Ng*, *N. gonorrhoeae *(AAW89271); *Nm*, *N. meningitidis *(NP284201); *Cv*, *Chromobacterium violaceum *(NP903735); *Pl*, *Photorhabdus luminescens *(NP927854); *Cj*, *Campylobacter jejuni*, (NP281793); *Cc*, *Campylobacter coli *(ZP00366779); *Cu*, *Campylobacter upsaliensis *(ZP00369961); *Hh*, *Helicobacter hepaticus *(NP860614); *Zm*, *Zymomonas mobilis *(1b, YP162179).

The sequences of the family 2 Ape proteins are highly homologous (Fig. 7) and, like the family 1a proteins, are characterized by having five distinct segments of high similarity separating the proposed catalytic motifs I and VII (Fig. 7). However, the consensus motifs identified are unique to this family (Fig. 7). Whereas the GDS(H/F) sequence is conserved in motif I, the conserved residues separating the catalytic D and H of consensus motif VII are distinctively G and I/V. As with the family 1 proteins, the family 2 sequences are encoded within the genomes of a number of important human pathogens, such as species of *Neisseria*, *Campylobacter *and *Helicobacter*.

**Figure 8:**
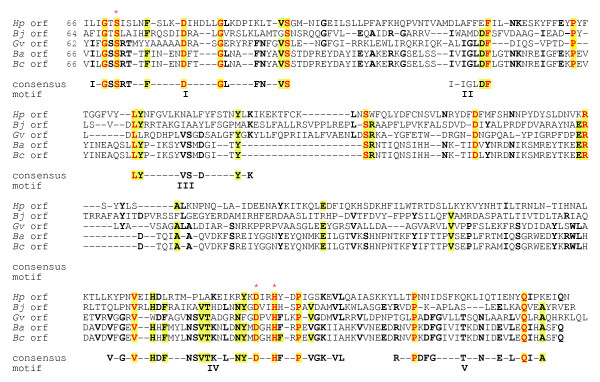
**Sequence alignment of family 3 *O*-acetylpeptidoglycan esterases (Ape3) and identification of consensus motifs**. The residues in bold face and yellow highlight denote greater than 50% and 80% identity, respectively, while those in red are invariant amongst all sequences. The numbers in brackets denote the length of intervening sequences and the potential catalytic residues are identified with the red asterisks. Abbreviations (accession numbers): *Hp*, *Helicobacter pylori *(NP207650); *Bj*, *Bradyrhizobium japonicum *(NP767014); *Gv*, *Gloeobacter violaceus *(NP923892); *Ba*, *Bacillus anthracis *(NP843402); *Bc*, *B. cereus *(NP977300).

The family 3 proteins have very little in common with either the family 1 or family 2 proteins (Fig. 6). In fact, the GDS(H/F) motif is replaced with G(T/S)S and there does not appear to be any consensus in residues separating the catalytic D and H residues of motif VII (Fig. 8). Whereas two other segments of some similarity are present between the catalytic motifs, there is a large region with minimal sequence similarity. This variation seen within the grouping suggests that further division of this family may occur once more genome sequences become available. Nonetheless, again this family does include sequences from some important pathogens such as *Helicobacter pylori *and *Bacillus anthracis*.

### Predicted cellular localization of Ape

The SignalP, PSORT and DAS prediction programs indicated that almost all of the hypothetical Pat and Ape protein sequences have an N-terminal localization signal. The only exception appears to be with the Ape sequence from *H. hepaticus*. Family 1 proteins from *H. hepaticus*, *P. luminescnes*, *Z. mobilis *and *P. gingivalis *may have a cytoplasmic membrane association due to the retention of an α-helical transmembrane anchor. However, the other Ape1 sequences contain a predicted signal peptidase I cleavage site that should release the expressed proteins from the cytoplasmic membrane. In the case of the Ape2 homologs, the *Campylobacter *sp. and *P. luminescens *sequences are predicted to associate with the cytoplasmic membrane, while the *Neisseria *sp. and *C. violaceum *proteins would be cleaved by signal peptidase I releasing them to the periplasm. The family 3 Ape from *H. pylori *is also predicted to remain associated with the cytoplasmic membrane. These predictions are thus consistent with the proposed function of the expressed proteins as participating in the *O*-acetylation of peptidoglycan as a maturation event occurring within the periplasm [5].

### Genomic organization of the OAP cluster

Despite their distant phylogenic relationship, the *patA *and *ape *genes are closely associated on respective chromosomes and comprise a small cluster we have called *O*-acetylpeptidoglycan (OAP) (Fig. 3). However, different gene organizations were observed which appear to be specified by the type of *ape *genes present. Thus, species containing the Ape1a proteins possess the strict gene order of *pat *immediately followed by *ape2 *and then *ape1a*. In contrast, the *ape1b *gene always appears to be located upstream of *pat*, sometimes with the addition of an intervening *ape1c *gene. The only *ape2 *not located downstream of *pat *is found in *Zymomonas mobilis *where instead it is positioned upstream together with an *ape1b *gene. All of the other *ape1b *genes are situated at the start of the OAP cluster and are followed in order by an *ape1c *and then *pat*.

Interestingly, the family 3 Ape appears to be capable of replacing the function(s) of both the family 1 and 2 proteins because its gene is situated alone immediately downstream of *pat*. Thus, this gene arrangement more closely resembles that of distantly related organisms like *Bacillus anthracis*, *Bradyrhizobium japonicum *and *Gloeobacter violaceus *than that of *H. hepaticus*.

### Extent of peptidoglycan *O*-acetylation in Pat- and Ape-producing bacteria

With the exception of *Agrobacterium tumefaciens*, none of the bacteria listed in Fig. 3 are xylanolytic suggesting that the discovered genes may be used by the respective bacteria for the modification of their peptidoglycans as proposed for *N. gonorrhoeae*. However, of these listed bacteria, only *N. gonorrhoeae *was previously known to *O*-acetylate its peptidoglycan [5,6,27]. Hence, we investigated the presence of this modification in a selection of the bacteria possessing the OAP cluster of genes.

Following the isolation and purification of the peptidoglycans from cultures of the respective cells grown to late exponential phase, the quantification of base-labile, ester-linked acetate was determined by the HPLC-based method previously described [27]. Of the nine species tested, eight were found to possess *O*-acetylated peptidoglycan (Table 1). The levels of this *O*-acetylation were not stoichiometric, consistent with previous observations made with each of the other known bacteria that modify their peptidoglycan in this manner [5,6,27]. Thus, the extent of *O*-acetylation ranged from 14.7 % to 65.6 % relative to muramic acid content. These data thus provide preliminary support for the ascribed function of the OAP cluster of genes for the *O*-acetylation of peptidoglycan. With *A. tumefaciens*, only minimal levels of peptidoglycan *O*-acetylation were detected suggesting that the "Ape" proteins produced by this xylanolytic bacterium may function as authentic xylan esterases. Alternatively, environmental signals may be required by this plant pathogen to induce the *O*-acetylation of its peptidoglycan.

## Conclusion

A new cluster of genes putatively involved in regulating O-linked acetate levels has been identified in a number of bacteria. These *O*-acetylpeptidoglycan clusters all consist of an *O*-acetyltransferase and at least one *O*-acetyl esterase. The *O*-acetyltransferases demonstrate high amino acid similarity to members of the MBOAT family of acetyltransferases [24] while the *O*-acetyl esterases share similarity to the CAZy family 3 esterases (CE3). The previously recognized members of family CE3 are all acetyl xylan esterases; no de-*N*-acetyltransferases belong to this family. Acetyl xylan esterases aid in the degradation of xylan by removing the blocking *O*-linked acetate and thereby permitting its subsequent hydrolysis by xylanases (reviewed in [9]). As many of the bacteria identified with these *O*-acetyl esterases are not xylanolytic and their genomes do not appear to encode xylanases, we postulated that these hypothetical esterases function on peptidoglycan to specifically catalyze de-*O*-acetylation. Consequently, they have been termed Ape proteins for their hypothetical *O*-acetylpeptidoglycan esterase activity.

Recognition of different domain structures for each of the Ape proteins prompted us to divide them into three distinct families. Of the families, only Ape3 appears to lack a clear conserved domain structure and this may represent a group that needs more members to permit its definitive subdivision. However, each of the bacteria possessing an Ape3 protein contain the unique cluster order of an *O*-acetyltransferase followed by a single *O*-acetyl esterase. The other Ape proteins are associated with an *O*-acetyltransferase, but always seem to be found in pairs consisting of Ape1b and Ape1c or Ape1a/1b and an Ape2. This ORF organization may translate into the evolutionary development of the Ape families. In fact, evolutionary divergence seems plausible for the Ape1 and Ape2 proteins, where duplication of an Ape1a/1b protein and divergence into an entirely new protein could have easily occurred. However, the ORF organization in *Helicobacter *species makes this theory far more confounded. Alternatively, differences between the Ape families may be linked to separate cellular functions. Some of the Ape proteins, especially those associated with either the cytoplasmic membrane and cytoplasm, may be involved in peptidoglycan recycling, while those in the periplasm and/or outer membrane of the gram-negative bacteria may be involved in the general maintenance of the peptidoglycan sacculus.

Regardless of their specific role in peptidoglycan metabolism, the activity of Ape proteins would be essential for the removal of acetate which would otherwise preclude the lysis of peptidoglycan by endogenous enzymes. Indeed, the major lytic enzymes associated with both the biosynthesis and maintenance of peptidoglycan are the lytic transglycosylases, enzymes that require a free C-6 hydroxyl group on muramoyl residues to produce their 1,6-anhydro product. Given that the bacteria harboring genes apparently involved with this activity were demonstrated to produce *O*-acetylated peptidoglycan, Ape activity would be required to function in advance of the lytic transglycosylase, many of which act processively along peptidoglycan strands [1], to provide appropriate sites for cleavage. Although strictly conjecture at this point, it is likely that these Ape proteins would be associated with the peptidoglycan 'replicase' holoenzyme complexes [1] which have been shown to include both the lytic transglycosylases and penicillin-binding proteins [28-30]. In this regard, it should be noted that many of the *ape *genes are in close proximity to other genes encoding enzymes involved in cell wall associated functions. For example, the three *Campylobacter *sp. contain a ferritin gene (*cft *and *pfr*) immediately upstream, while both *C. coli *and *C. upsaliens *also possess genes encoding a potential ABC transporter and an outer membrane protein directly downstream of the OAP cluster. Similarly, both *Bacteroides *sp. encode a putative cation-efflux transporter upstream of their OAP clusters, while the *Z. mobilis *chromosome encodes a nearby cell wall hydrolase. Hence, through a dynamic process of removal and attachment of *O*-acetate, the bacteria would be able to obtain a high level of localized control over the degradation of peptidoglycan. This would facilitate, for example, the efficient insertion of pores and flagella, localize spore formation, and control the level of general peptidoglycan turnover.

It is difficult to determine whether or not a correlation exists between *O*-acetylation levels and the type or number of *O*-acetyl esterases present in a particular organism. In the case of the Ape1b/1c and Ape3 this may be because so few organisms have been tested for peptidoglycan *O*-acetylation. However, even *Neisseria *sp., *C. violaceum*, and the *Campylobacter *sp., which all possess Ape1a and Ape2, contain *O*-acetylation levels that vary significantly. This suggests that the level of *O*-acetylpeptidoglycan esterase activity may be regulated by compartmentalization or at the level of transcription/translation. The Ape family 1a/1b proteins are different from the Ape1c proteins due to the presence of an extra amino acid segment. The variation in each of these segments raises questions of substrate specificity and function that must be proven, but it is interesting to note that one of the segments has partial similarity to PBP 4, a D,D carboxypeptidase. These proteins are peptidoglycan maintenance proteins, and therefore suggest that the extra protein segment found in the Ape1a/b proteins may be a peptidoglycan specific fragment or one directing protein-protein interactions. This would certainly explain its absence from the *O*-acetylxylan esterase CE3 family, but not necessarily from the other Ape families.

Two models of peptidoglycan *O*-acetylation have been proposed in a recent review [6]. The first model involves the sequential action of two proteins. The first protein would be located within the cytoplasmic membrane and facilitates the transfer of acetate from acetyl-CoA to the periplasm, where a second protein accepts the acetate and attaches it to peptidoglycan. The second model consists of a single membrane bound *O*-acetyltransferase that performs both the transport of acetate across the cytoplasmic membrane and the peptidoglycan *O*-acetylation steps. A peptidoglycan *O*-acetyltransferase recently identified in *S. aureus *[17] is expected to act by the second peptidoglycan *O*-acetylation pathway. This *O*-acetyltransferase bears no resemblance to the *O*-acetyltransferases identified in the present study. This strongly suggests that, as with the *O*-acetyl esterases together with the lytic transglycosylases [31] and the PBPs [32], different classes of peptidoglycan *O*-acetyltransferases exist.

In summary, experiments are still required to conclusively demonstrate that the *O*-acetyltransferases and Ape proteins identified in this study are actually involved in controlling peptidoglycan *O*-acetylation. However, it is important to note that all the organisms that demonstrated some level of peptidoglycan *O*-acetylation also contain the putative *O*-acetylation cluster. Verification of peptidoglycan *O*-acetylation in the other bacteria containing the *O*-acetylation cluster are in progress, and should further strengthen the link between these two events. In addition, experiments characterizing the gene product of *N. gonorrhoeae *Ape1a have been initiated and preliminary evidence supports its function as an *O*-acetylpeptidoglycan esterase.

## Methods

### Chemicals and reagents

DNase I, RNase A, and pronase were purchased from Roche Molecular Biochemicals (Laval, PQ), while Fisher Scientific (Nepean, ON), provided Luria Bertani (LB) growth medium. All other growth media were purchased from Difco Laboratories (Detroit, MI), and all other chemicals and reagents were from Sigma Chemical Co. (St. Louis, MO).

### Bacterial strains and growth media

*Agrobacterium tumefaciens *and *Photorhabdus luminescens *were both cultured in Tryptic Soy Broth (TSB) for 48 h at ambient temperature with shaking (200 rpm) while *Bacillus cereus *was grown in Penassay Broth as previously described [33]. *Chromobacterium violaceum *was cultured in LB broth with shaking (200 rpm) for 48 h at ambient temperature. *Bacteroides fragilis and B. thetaiotaomicron *were grown at 37°C under strict anaerobic conditions using *Bacteroides *Bile Esculin Broth (BBE) supplemented with 100 mg/mL gentamicin while *Ruminococcus flavefaciens *was cultured anaerobically using Dehority's Complete Artificial Medium as previously described [34]. *Helicobacter pylori *and *Campylobacter jejuni *were cultured at 37°C under microaerophilic conditions (10% (vol/vol) O_2_, 5% (vol/vol) CO_2_, and 85% (vol/vol) N_2_) in Brain Heart Infusion Broth supplemented with 10% irradiated horse serum and TSB supplemented with 0.6% (w/vol) yeast extract, respectively. *Bradyrhizobium japonicum *was grown in Yeast Extract Mannitol broth at ambient temperature as previously described [35].

### Peptidoglycan isolation and determination of extent of O-acetylation

*O*-Acetylated peptidoglycan was isolated and purified from the various bacteria, taking the necessary precautions to prevent the facile hydrolysis of *O*-acetyl groups as previously described [36]. Quantification of *O*-acetyl content was performed by both the HPLC-based organic acid analysis using an Aminex HPX-87H Bio-Rad column as previously described [27] and using the Megazyme Acetic Acid Assay kit (Megazyme International Ireland Ltd., Wicklow, Ireland). The extent of *O*-acetylation is presented as a percentage of muramic acid content as determined by quantitative aminosugar analysis [37].

### Identification and genomic analysis of a putative cluster of peptidoglycan *O*-acetylation ORFs

The raw nucleotide sequence data of the *N. gonorrhoeae *FA1090 genome project [18] was searched for hypothetical proteins involved in acetate transport using the *Pseudomonas aeruginosa *protein sequence for AlgI as the probe (*algI *encodes a putative acetate translocator involved with the biosynthesis of alginate [14,15]). Identification of open reading frames, protein translations, and isoelectric points (pI) were performed using Clone Manager 5. Signal sequence and cellular localization predictions were performed using signalP version 3 [38], PSORT [39], and the DAS Transmembrane Prediction Server [40]. Searches for homologs to the characterized *N. gonorrhoeae *ORFs in the NCBI database were carried out using the BLASTP search engine to tentatively identify functions for each of the ORFs in the cluster[41]. Sequences with an (E) value threshold of 1e-06 were used to generate multiple alignments using ClustalW Version 1.8 software [42]. Manual correction of these alignments was performed based on the original BLASTP results in conjunction with the MEME/ MAST Version 3.0 motif discovery tool [43]. Predictions of secondary structure were performed using the GOR IV Secondary Structure Prediction method [44] and PSI PRED [45], while searches for known domains were performed using CD-Search [46] at NCBI.

## Authors' contributions

JTW and AJC conceived the study, participated in the sequence alignments and analyses, and wrote the manuscript. JMP performed the quantification of peptidoglycan *O*-acetylation, and all authors read and approved the final manuscript.

## Figures and Tables

**Table 1:** Extent of peptidoglycan *O*-acetylation in bacteria possessing OAP gene clusters

**Species**	**Strain**	**% *O*-Acetylation**^1^
*Agrobacterium tumefaciens*	A6	3.44 ± 0.79
	371	1.52 ± 0.36
*Bacillus cereus*	ATCC 10702	24.7 ± 0.09
*Bacteroides fragilis*	ATCC 25285	69.4 ± 1.2
*Bacteroides thetaiotamicron*	ATCC 29741	27.0 ± 1.2
*Bradyrhizobium japonicum*	532 C	18.5 ± 2.2
*Campylobacter jejuni*	ATCC 700819	62.7 ± 5.6
	NCTC 11168	55.7 ± 1.4
*Chromobacterium violaceum*	ATCC 12472	14.7 ± 2.5
*Helicobacter pylori*	ATCC 700392	45.6 ± 3.2
*Photorhabdus luminescens*	ATCC 29999	65.6 ± 1.6
*Ruminococcus flavefaciens*	007	33.4 ± 3.4

1. Average ± s.d.(n = 3)of base-labile acetate content relative to muramic acid concentrations in isolated and purified peptidoglycan.
